# The endoplasmic reticulum–localized enzyme zDHHC6 mediates S-acylation of short transmembrane constructs from multiple type I and II membrane proteins

**DOI:** 10.1016/j.jbc.2023.105201

**Published:** 2023-09-01

**Authors:** Christine Salaun, Nicholas C.O. Tomkinson, Luke H. Chamberlain

**Affiliations:** 1 Strathclyde Institute of Pharmacy and Biomedical Sciences, University of Strathclyde, Glasgow, United Kingdom; 2 Department of Pure and Applied Chemistry, University of Strathclyde, Glasgow, United Kingdom

**Keywords:** S-acylation, palmitoylation, zDHHC enzymes, zDHHC6, transmembrane, mPEG-Click, endoplasmic reticulum

## Abstract

In this study, we investigated the S-acylation of two host cell proteins important for viral infection: TMPRSS2 (transmembrane serine protease 2), which cleaves severe acute respiratory syndrome coronavirus 2 spike to facilitate viral entry, and bone marrow stromal antigen 2, a general viral restriction factor. We found that both proteins were S-acylated by zDHHC6, an S-acyltransferase enzyme localized at the endoplasmic reticulum, in coexpression experiments. Mutagenic analysis revealed that zDHHC6 modifies a single cysteine in each protein, which are in proximity to the transmembrane domains (TMDs). For TMPRSS2, the modified cysteine is positioned two residues into the TMD, whereas the modified cysteine in bone marrow stromal antigen 2 has a cytosolic location two amino acids upstream of the TMD. Cysteine swapping revealed that repositioning the target cysteine of TMPRSS2 further into the TMD substantially reduced S-acylation by zDHHC6. Interestingly, zDHHC6 efficiently S-acylated truncated forms of these proteins that contained only the TMDs and short juxtamembrane regions. The ability of zDHHC6 to modify short TMD sequences was also seen for the transferrin receptor (another type II membrane protein) and for five different type I membrane protein constructs, including cluster of differentiation 4. Collectively, the results of this study show that zDHHC6 can modify diverse membrane proteins (type I and II) and requires only the presence of the TMD and target cysteine for efficient S-acylation. Thus, zDHHC6 may be a broad specificity S-acyltransferase specialized for the modification of a diverse set of transmembrane proteins at the endoplasmic reticulum.

S-acylation is a widespread post-translational modification of soluble and transmembrane proteins affecting up to 10% of the proteome (1, 2, 3). This process, involving the attachment of fatty acyl chains onto cysteine residues, regulates the localization, stability, interactions, and functions of a diverse array of cellular proteins. S-acylation is a reversible modification that is regulated by the opposing actions of zDHHC S-acyltransferase (on) and thioesterase enzymes (off) (4, 5). zDHHC enzymes mediate S-acylation through a two-step process that involves autoacylation of the active site cysteine (DHHC motif) followed by transfer of the acyl chain to a cysteine residue of a substrate protein (6, 7, 8). There are 23 human zDHHC enzymes, all of which are predicted transmembrane proteins that localize predominantly to the endoplasmic reticulum (ER) and Golgi (9, 10).

A major outstanding question in the S-acylation field is the substrate networks of individual zDHHC enzymes and how enzyme–substrate specificity is encoded. We have previously investigated the S-acylation of soluble S-acylated proteins including SNAP25, cysteine-string protein (CSP), and the Sprouty/SPRED family (11, 12, 13). Through the analysis of these substrates, we were able to classify a subset of Golgi enzymes as high activity/low specificity and low activity/high specificity (14). zDHHC3 and zDHHC7 are the archetypal high-activity/low-specificity enzymes, which mediate the S-acylation of a diverse array of cellular proteins in the apparent absence of any conserved substrate recognition motifs or detectable interaction. In this case, we proposed that the enzymes exist in a high-activity state that allows them to transfer their acyl chain onto any appropriately positioned and reactive cysteine at the membrane interface. Specificity in this case is encoded by cysteine position/reactivity more than specific enzyme–substrate recognition mechanisms. In contrast, zDHHC17 is an archetypal low-activity/high-specificity enzyme, which requires a specific means of substrate recognition/recruitment to facilitate S-acylation. For zDHHC17, this substrate recognition involves binding of an ankyrin repeat domain on the enzyme to a conserved zDABM sequence in the substrate or by a recently described novel binding mechanism independent of the ankyrin-repeat domain (15, 16, 17). We propose that this more selective recognition and S-acylation mediated by low-activity/high-specificity enzymes might be particularly important for the recruitment and S-acylation of soluble proteins. It is currently unclear whether other membrane compartments also contain a combination of high- and low-specificity enzymes.

In this study, we have explored the S-acylation of transmembrane proteins, focusing on two proteins that are involved in viral infection. Transmembrane serine protease 2 (TMPRSS2) is a protease mediating the cleavage of viral envelope and spike proteins of various viruses (including influenza (18) and severe acute respiratory syndrome coronavirus 2 [SARS-CoV-2] (19)). TMPRSS2 facilitates virus entry into host cells, making it a potential drug target for combating viral infections. TMPRSS2 has also been associated with physiological processes, such as digestion, blood coagulation, inflammation, and tumor spreading (20). Bone marrow stromal antigen 2 (BST2)/tetherin/cluster of differentiation 317 (CD317), on the other hand, is an interferon-induced viral restriction factor whose broad activity is counteracted by many viruses (21). BST2 can restrict virion release (22) and is an activator of the NF-κB pathway (23). These two proteins have a single type II transmembrane domain (TMD) and have been identified as S-acylated proteins in proteomic studies (swisspalm.org; #O15393 and #Q10589). Type II membrane proteins have a single membrane-spanning domain with their N terminus in the cytosol, whereas type I membrane proteins have their N terminus translocated into the ER lumen during biosynthesis. Through coexpression studies, click chemistry, and mutagenic analyses, we show that these proteins are S-acylated by zDHHC6 on cysteine residues in proximity to their TMDs. Furthermore, by analysis of other TMD proteins, we propose that zDHHC6 is a broad specificity S-acyltransferase that can mediate S-acylation of cysteine residues proximal to TMDs in multiple proteins during their transit through the ER.

## Results

### BST2 and TMPRSS2 are S-acylated by specific zDHHC enzymes including zDHHC6

To investigate the S-acylation of TMPRSS2 and BST2, we used a click-PEG methodology recently developed by our group (24). In this assay, cells are incubated with azide-C16:0 overnight, which is incorporated into S-acylated proteins. Cell lysates are then incubated with alkyne-PEG (5 kDa), and the click reaction of the azide and alkyne tags leads to a molecular mass increase in S-acylated proteins that can be visualized by immunoblotting.

**Figure 1:**
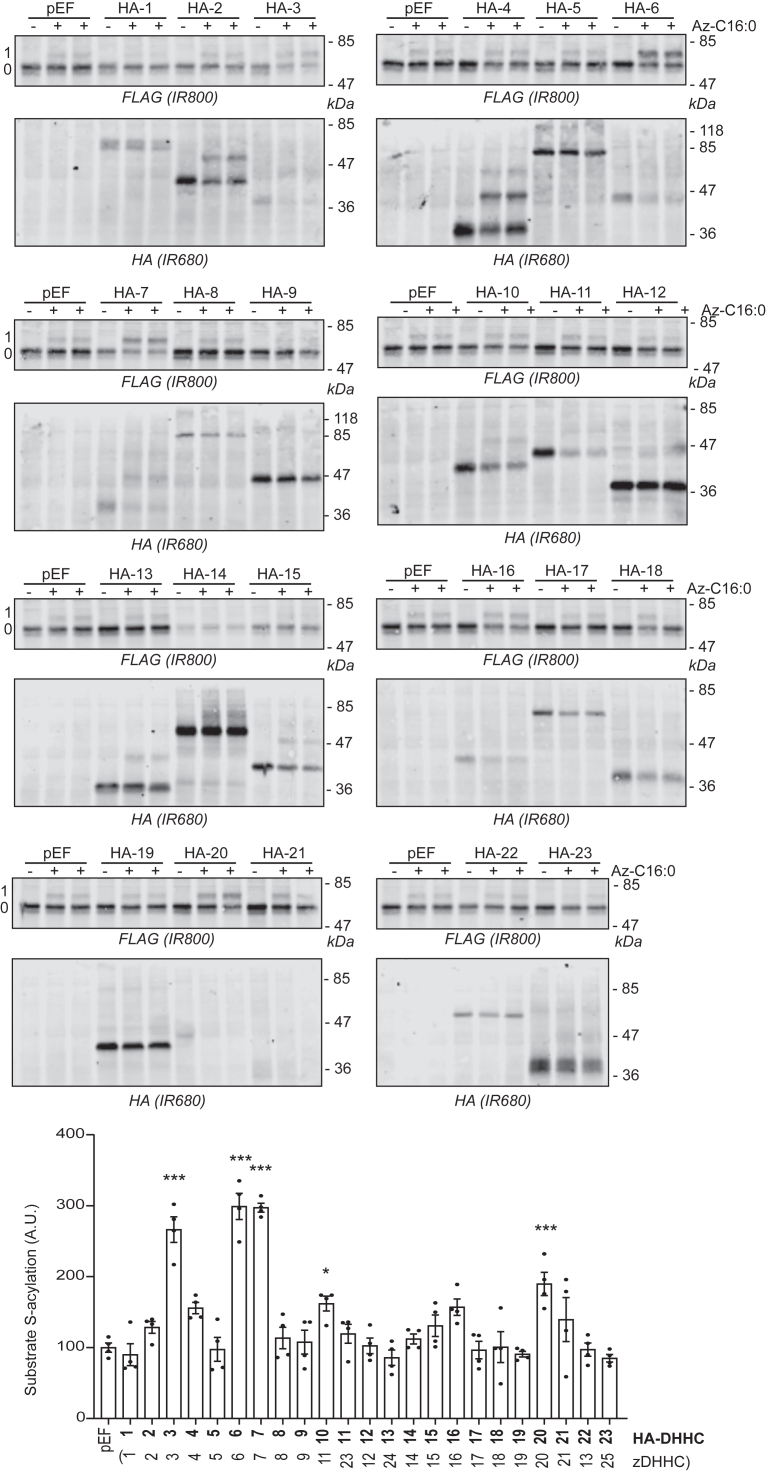
**Identification of TMPRSS2 S-acylating enzymes.** HEK293T cells were cotransfected with a plasmid encoding TMPRSS2-FLAG together with a plasmid encoding HA-tagged isoforms of mouse DHHC (HA-1 to HA-23) (4) or the empty plasmid (pEF). After 6 h, cells were metabolically labeled overnight with either palmitate (−Az-C16:0) or palmitate azide (+Az-C16:0) followed by click chemistry using alkyne-PEG. Proteins were resolved by SDS-PAGE and transferred to nitrocellulose membranes that were probed first with an anti-FLAG antibody (revealed with secondary antibodies coupled to the infrared dye IR800) followed by an anti-HA antibody (and IR680-coupled secondary antibodies). Positions of molecular weight markers are shown on the *right-handside* of the membranes, whereas numbers on the *left* (0 and 1) relate to the number of modified cysteines within the substrate. The *graph* at the *bottom* of the figure shows mean ± SEM of normalized substrate S-acylation by each HA-tagged enzyme; *filled circles* represent individual samples (n = 4 different cell samples from two independent experiments). The correspondence with the current up-to-date nomenclature (zDHHC-1 to zDHHC-25) is also indicated at the *bottom* of the *graph* between *brackets*. Statistical analysis (ANOVA followed by a Dunnett’s *post hoc* test) was performed to reveal significant S-acylation of the substrate by the exogenously expressed enzymes *versus* its acylation by endogenous enzymes (pEF samples) (∗*p* < 0.05; ∗∗∗*p* < 0.001). HA, hemagglutinin tag; HEK293T, human embryonic kidney 293T cell line; TMPRSS2, transmembrane serine protease 2.

**Figure 2:**
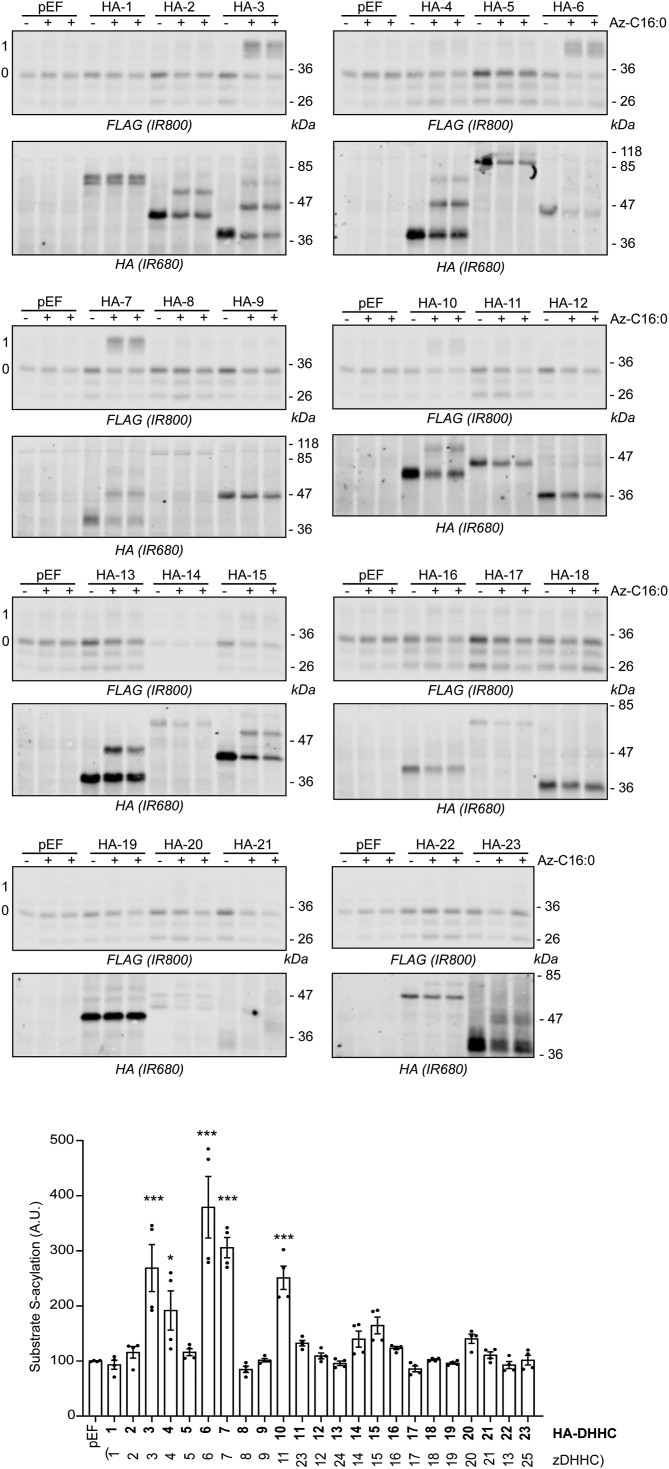
**Identification of BST2 S-acylating enzymes.** HEK293T cells were cotransfected with a plasmid encoding FLAG-BST2 together with a plasmid encoding HA-tagged isoforms of mouse DHHC (HA-1 to HA-23) or the empty plasmid (pEF). After 6 h, cells were metabolically labeled overnight with either palmitate (−Az-C16:0) or palmitate azide (+Az-C16:0) followed by click chemistry using alkyne-PEG. Proteins were resolved by SDS-PAGE and transferred to nitrocellulose membranes that were probed first with an anti-FLAG antibody (revealed with secondary antibodies coupled to the infrared dye IR800) followed by an anti-HA antibody (and IR680-coupled secondary antibodies). Positions of molecular weight markers are shown on the *right-handside* of the membranes, whereas numbers on the *left* (0 and 1) relate to the number of modified cysteines within the substrate. The *graph* at the *bottom* of the figure shows mean ± SEM of normalized substrate S-acylation by each HA-tagged enzyme; *filled circles* represent individual samples (n = 4 different cell samples from two independent experiments). The correspondence with the current up-to-date nomenclature (zDHHC-1 to zDHHC-25) is also indicated at the *bottom* of the *graph* between *brackets*. Statistical analysis (ANOVA followed by a Dunnett’s *post hoc* test) was performed to reveal significant S-acylation of the substrate by the exogenously expressed enzymes *versus* its acylation by endogenous enzymes (pEF samples) (∗*p* < 0.05; ∗∗∗*p* < 0.001). BST2, bone marrow stromal antigen 2; HA, hemagglutinin tag; HEK293T, human embryonic kidney 293T cell line.

Human embryonic kidney 293T (HEK293T) cells were transfected with FLAG-tagged TMPRSS2 or BST2 together with plasmids encoding 23 different hemagglutinin (HA)-tagged zDHHC enzymes. Following cell labeling and click chemistry, cell lysates were resolved by SDS-PAGE and transferred to nitrocellulose for immunoblotting analysis using anti-FLAG and anti-HA antibodies. The results for TMPRSS2 are shown in Figure 1: in addition to zDHHC3 and zDHHC7 (high-activity/low-specificity enzymes), cotransfection with zDHHC6 also led to a pronounced significant increase in TMPRSS2 S-acylation. The results with BST2 (Fig. 2) were similar, with zDHHC6 also stimulating a marked increase in the S-acylation of this protein. As with TMPRSS2, zDHHC3 and zDHHC7 also promoted an increase in BST2 S-acylation. Other enzymes that led to a significant increase in S-acylation of TMPRSS2 and BST2 included zDHHC11 (HA-DHHC10) (both TMPRSS2 and BST2), zDHHC20 for TMPRSS2, and zDHHC4 for BST2. For both proteins, zDHHC6 gave the highest mean increase in S-acylation.

### Identification of S-acylated cysteines in TMPRSS2 and BST2

The zDHHC enzyme screens shown in Figures 1 and 2 revealed that TMPRSS2 and BST2 are S-acylated to the highest level by zDHHC6. Although zDHHC3 and zDHHC7 also led to a similar magnitude increase in S-acylation, we and others have previously shown that these enzymes are active against most substrate proteins in coexpression experiments with no obvious substrate selectivity. Therefore, for the remainder of this study, we focused on zDHHC6 with the aim of determining how this enzyme recognizes TMPRSS2 and BST2 as substrates for S-acylation. There is currently no published study examining zDHHC6 substrate recognition.

**Figure 3:**
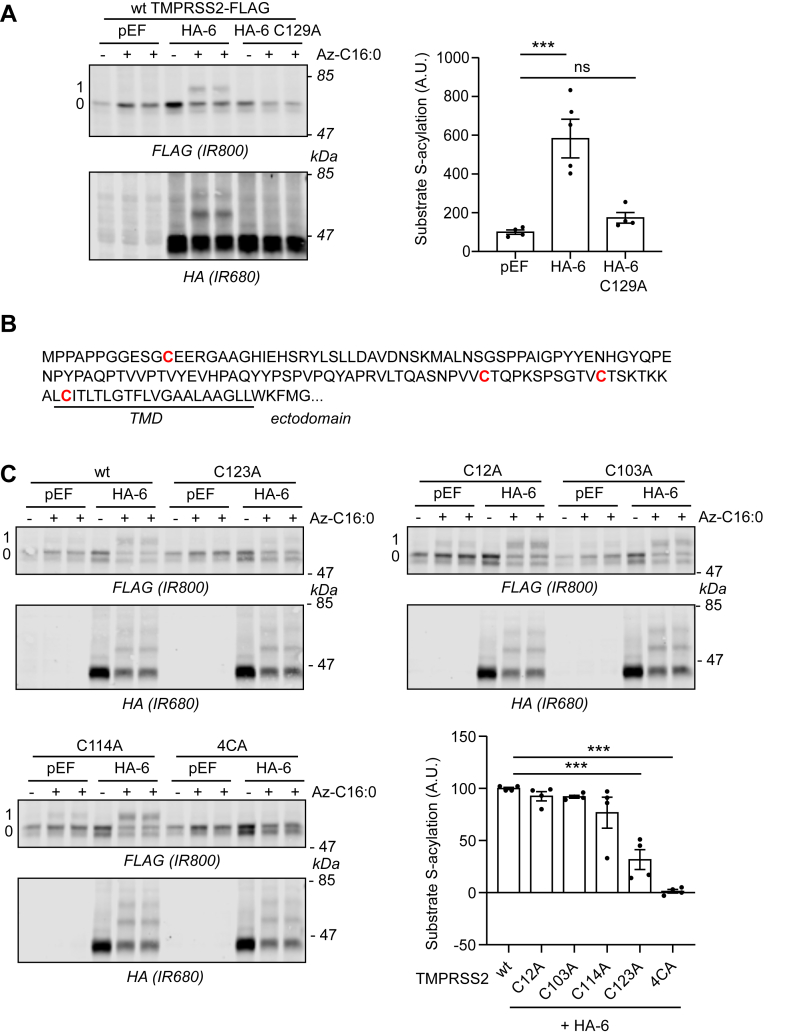
**Identification of the transmembrane cysteine C123 as the main zDHHC6-mediated S-acylated cysteine within TMPRSS2.***A*, HEK293T cells were cotransfected with a plasmid encoding TMPRSS2-FLAG together with a plasmid encoding HA-tagged isoforms of either mouse DHHC6 (HA-6) or its catalytically inactive form (HA-6 C129A) or the empty plasmid (pEF). Six hours post-transfection, cells were metabolically labeled overnight with either palmitate (−Az-C16:0) or palmitate azide (+Az-C16:0) followed by click chemistry using alkyne-PEG. Proteins were resolved by SDS-PAGE and transferred to nitrocellulose membranes. The membranes were probed first with an anti-FLAG antibody (revealed with secondary antibodies coupled to the infrared dye IR800) followed by an anti-HA antibody (and IR680-coupled secondary antibodies). Positions of molecular weight markers are shown on the *right-hand side* of the membranes, whereas numbers on the *left* (0 and 1) relate to the number of modified cysteines within TMPRSS2. The *graph* on the *right-hand side* of the *panel* shows mean ± SEM of normalized substrate S-acylation by HA-tagged enzymes; *filled circles* represent individual samples (n = 4 different cell samples from two independent experiments). Statistical analysis (ANOVA followed by a Dunnett’s *post hoc* test) was performed to reveal significant S-acylation of the substrate following the expression of exogenous HA-DHHC enzymes *versus* its acylation by endogenous enzymes (pEF samples) (∗∗∗*p* < 0.001; ns, *p* > 0.05). *B*, partial amino acid sequence of TMPRSS2: cytosolic N-terminal domain of TMPRSS2 and transmembrane domain (TMD; *underlined*). The four cysteines are highlighted in *bold* and *red*. *C*, HEK293T cells were cotransfected with a plasmid encoding either wt TMPRSS2-FLAG or cysteine to alanine mutants (four single mutants: C12A, C103A, C114A, and C123A, and a quadruple mutant [4CA]: C12_C103_C114_C123A) together with a plasmid encoding HA-DHHC6 (HA-6) or the empty control plasmid (pEF). Six hours post-transfection, cells were metabolically labeled overnight with either palmitate (−Az-C16:0) or palmitate azide (+Az-C16:0) followed by click chemistry using alkyne-PEG. Proteins were resolved by SDS-PAGE and transferred to nitrocellulose membranes. The membranes were probed first with an anti-FLAG antibody (revealed with secondary antibodies coupled to the infrared dye IR800) followed by an anti-HA antibody (and IR680-coupled secondary antibodies). Positions of molecular weight markers (in kilodalton) are shown on the *right-hand side* of the membranes, whereas numbers on the *left* (0 and 1) relate to the number of modified cysteines within TMPRSS2. The *graph* on the *bottom right* of the *panel* shows mean ± SEM of normalized substrate S-acylation by HA-DHHC6 for each wt or mutant TMPRSS2 substrate. The mean value of the S-acylation mediated by the endogenous enzymes (*i.e.*, with pEF) was subtracted from the values of the specific HA-DHHC6-mediated S-acylation. *Filled circles* represent individual samples (n = 4 cell samples from two independent experiments). Statistical analysis (ANOVA followed by a Dunnett’s *post hoc* test) was performed to reveal significant differences between the S-acylation of the cysteine mutant substrates by HA-DHHC6 *versus* the S-acylation of wt TMPRSS2 (∗∗∗*p* < 0.001). HA, hemagglutinin tag; HEK293T, human embryonic kidney 293T cell line; ns, not significant; TMPRSS2, transmembrane serine protease 2.

**Figure 4:**
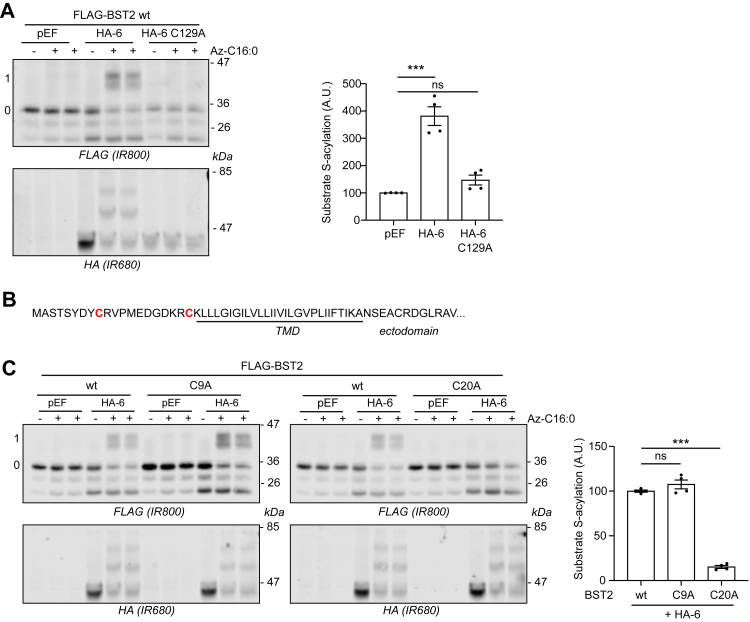
**Identification of the transmembrane-proximal cysteine C20 as the main zDHHC6-mediated S-acylated cysteine within BST2.***A*, HEK293T cells were cotransfected with a plasmid encoding FLAG-BST2 together with a plasmid encoding HA-tagged isoforms of either mouse zDHHC6 (HA-6) or its catalytically inactive form (HA-6 C129A) or the empty control plasmid (pEF). Six hours post-transfection, cells were metabolically labeled overnight with either palmitate (−Az-C16:0) or palmitate azide (+Az-C16:0) followed by click chemistry using alkyne-PEG. Proteins were resolved by SDS-PAGE and transferred to nitrocellulose membranes. The membranes were probed first with an anti-FLAG antibody (revealed with secondary antibodies coupled to the infrared dye IR800) followed by an anti-HA antibody (and IR680-coupled secondary antibodies). Positions of molecular weight markers are shown on the *right-hand side* of the membranes, whereas numbers on the *left* (0 and 1) relate to the number of modified cysteines within BST2. The *graph* on the *right-hand side* of the *panel* shows mean ± SEM of normalized substrate S-acylation by HA-tagged enzymes; *filled circles* represent individual samples (n = 4 different cell samples from two independent experiments). Statistical analysis (ANOVA followed by a Dunnett’s *post hoc* test) was performed to reveal significant S-acylation of the substrate following the expression of exogenous HA-DHHC enzymes *versus* its acylation by endogenous enzymes (pEF samples) (∗∗∗*p* < 0.001; ns, *p* > 0.05). *B*, partial amino acid sequence of BST2: cytosolic N-terminal domain of BST2 and transmembrane domain (TMD; *underlined*). The two cytosolic cysteines are highlighted in *bold* and *red*. *C*, HEK293T cells were cotransfected with a plasmid encoding either wt FLAG-BST2 or cysteine to alanine mutants (C9A and C20A) together with a plasmid encoding HA-DHHC6 (HA-6) or the empty control plasmid (pEF). Six hours post-transfection, cells were metabolically labeled overnight with either palmitate (−Az-C16:0) or palmitate azide (+Az-C16:0) followed by click chemistry using alkyne-PEG. Proteins were resolved by SDS-PAGE and transferred to nitrocellulose membranes. The membranes were probed first with an anti-FLAG antibody (revealed with secondary antibodies coupled to the infrared dye IR800) followed by an anti-HA antibody (and IR680-coupled secondary antibodies). Positions of molecular weight markers (in kilodalton) are shown on the *right-hand side* of the membranes, whereas numbers on the *left* (0 and 1) relate to the number of modified cysteines within BST2. The *graph* on the *right* of the *panel* shows mean ± SEM of normalized substrate S-acylation by HA-DHHC6 for each wt or mutant BST2 substrate. The mean value of the S-acylation mediated by the endogenous enzymes (*i.e.*, with pEF) was subtracted from the values of the specific HA-DHHC6-mediated S-acylation. *Filled circles* represent individual samples (n = 4 cell samples from two independent experiments). Statistical analysis (ANOVA followed by a Dunnett’s *post hoc* test) was performed to reveal significant differences between the S-acylation of the cysteine mutant substrates by HA-DHHC6 *versus* the S-acylation of wt BST2 (∗∗∗*p* < 0.001; ns, *p* > 0.05). BST2, bone marrow stromal antigen 2; HA, hemagglutinin tag; HEK293T, human embryonic kidney 293T cell line; ns, not significant.

S-acylation of both TMPRSS2 and BST2 was dependent on the catalytic cysteine of zDHHC6 (Figs. 3*A* and 4A), ruling out indirect effects of zDHHC6 coexpression on S-acylation of these proteins.

To identify the cysteine(s) in TMPRSS2 and BST2 that are targets of zDHHC6, mutant forms of the proteins were studied. As only a single prominent band shift was seen in the click-PEG experiments (Figs. 1 and 2), this suggests that each protein is likely modified on only a single cysteine. TMPRSS2 contains four cysteines in its cytoplasmic/transmembrane region (Fig. 3*B*). Each cysteine was therefore substituted to an alanine and S-acylation by zDHHC6 examined in coexpression assays in HEK293T cells. Figure 3*C* shows that removal of cysteine-12, -103, or -114 in TMPRSS2 had no significant effect on S-acylation. In contrast, cysteine-123 led to a marked loss of S-acylation. C123 is located two amino acids into the TMD of TMPRSS2 (Fig. 3*B*). For BST2 (which contains two cysteines in its cytoplasmic domain; Fig. 4*B*), removal of cysteine-9 had no effect on S-acylation, whereas a C20A substitution led to a complete loss of S-acylation (Fig. 4*C*). This cysteine is located at the interface of the TMD and cytosolic region of BST2 (Fig. 4*B*).

### S-acylation of TMPRSS2 by zDHHC6 depends on the position of the cysteine within the TMD

**Figure 5:**
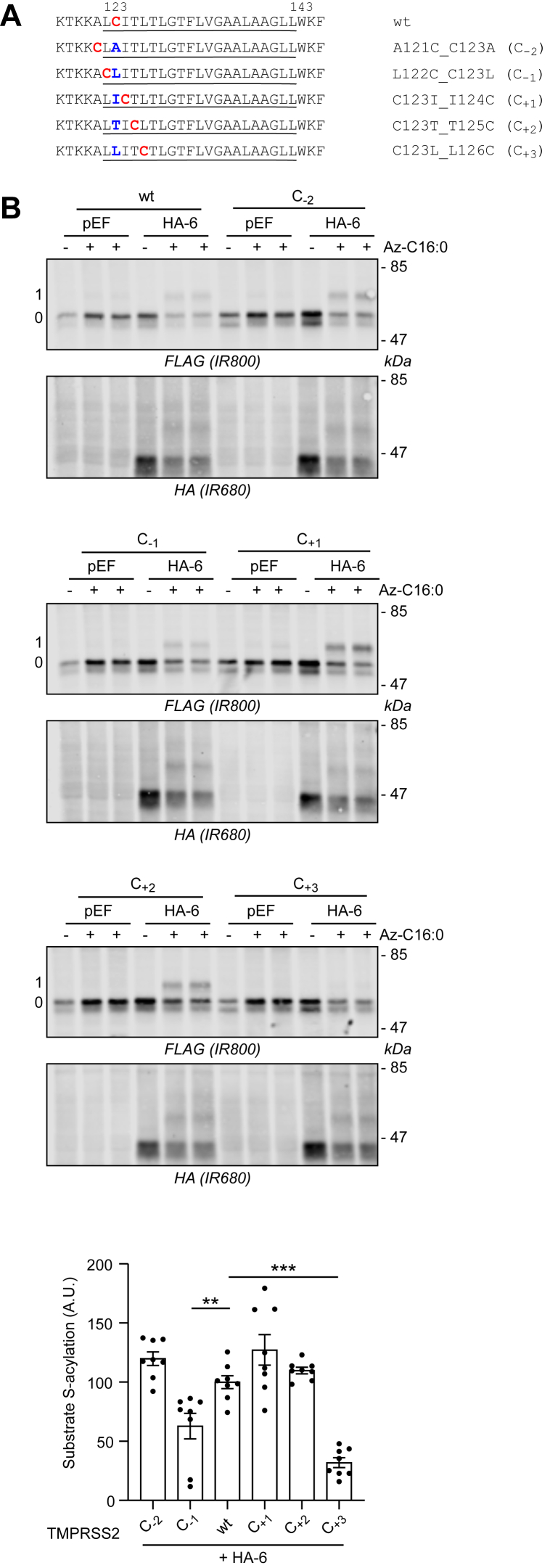
**Cysteine swapping within full-length TMPRSS2.***A*, schematic of the mutants of TMPRSS2-FLAG used in the analysis. Cysteine 123 (*in red and bold*) was swapped with the corresponding amino acid (*in blue and bold*) at positions 121, 122, 124, 125, and 126 within the transmembrane domain (*underlined*) of TMPRSS2. This results in the shifting of the cysteine between position −2 (C_−2_) and position +3 (C_+3_) relative to the position of the cysteine (position 0) within the wt protein. *B*, HEK293T cells were cotransfected with a plasmid encoding either wt TMPRSS2-FLAG or the mutants described in *A* together with a plasmid encoding HA-DHHC6 (HA-6) or the empty control plasmid (pEF). Six hours post-transfection, cells were metabolically labeled overnight with either palmitate (−Az-C16:0) or palmitate azide (+Az-C16:0) followed by click chemistry using alkyne-PEG. Proteins were resolved by SDS-PAGE and transferred to nitrocellulose membranes. The membranes were probed first with an anti-FLAG antibody (revealed with secondary antibodies coupled to the infrared dye IR800) followed by an anti-HA antibody (and IR680-coupled secondary antibodies). Positions of molecular weight markers (in kilodalton) are shown on the *right-hand side* of the membranes, whereas numbers on the *left* (0 and 1) relate to the number of modified cysteines within TMPRSS2. The *graph* at the *bottom* of the *panel* shows mean ± SEM of normalized substrate S-acylation by HA-DHHC6 for each wt or mutant TMPRSS2 substrate. The mean value of the S-acylation mediated by the endogenous enzymes (*i.e.*, with pEF) was subtracted from the values of the specific HA-DHHC6-mediated S-acylation. *Filled circles* represent individual samples (n = 8 cell samples from three independent experiments). Statistical analysis (ANOVA followed by a Dunnett’s *post hoc* test) was performed to reveal significant differences between the S-acylation of the cysteine mutant substrates by HA-DHHC6 *versus* the S-acylation of wt TMPRSS2 (∗∗∗*p* < 0.001; ∗∗*p* < 0.01). HA, hemagglutinin tag; HEK293T, human embryonic kidney 293T cell line; TMPRSS2, transmembrane serine protease 2.

To examine if S-acylation by zDHHC6 is dependent upon the exact position of the modified cysteine, we focused on TMPRSS2. A series of mutants were constructed in which the modified cysteine was moved either toward the cytoplasmic region or deeper into the membrane region (Fig. 5*A*). As shown in Figure 5*B*, zDHHC6 still S-acylated constructs in which the cysteine was moved toward the cytoplasmic region (A121C_C123A and L122C_C123L). In addition, zDHHC6 S-acylated mutants in which the cysteine was moved to positions 3 and 4 of the TMD (C123I_I124C and C123T_T125C). However, when the target cysteine was moved to position 5 of the TMD (C123L_L126C), S-acylation by zDHHC6 was substantially reduced (Fig. 5*B*). S-acylation was also reduced for L122C_C123L mutant although not at the same level as the C123L_L126C mutant.

### S-acylation of TMPRSS2 and BST2 by zDHHC6 requires only the TMDs and target cysteines

**Figure 6:**
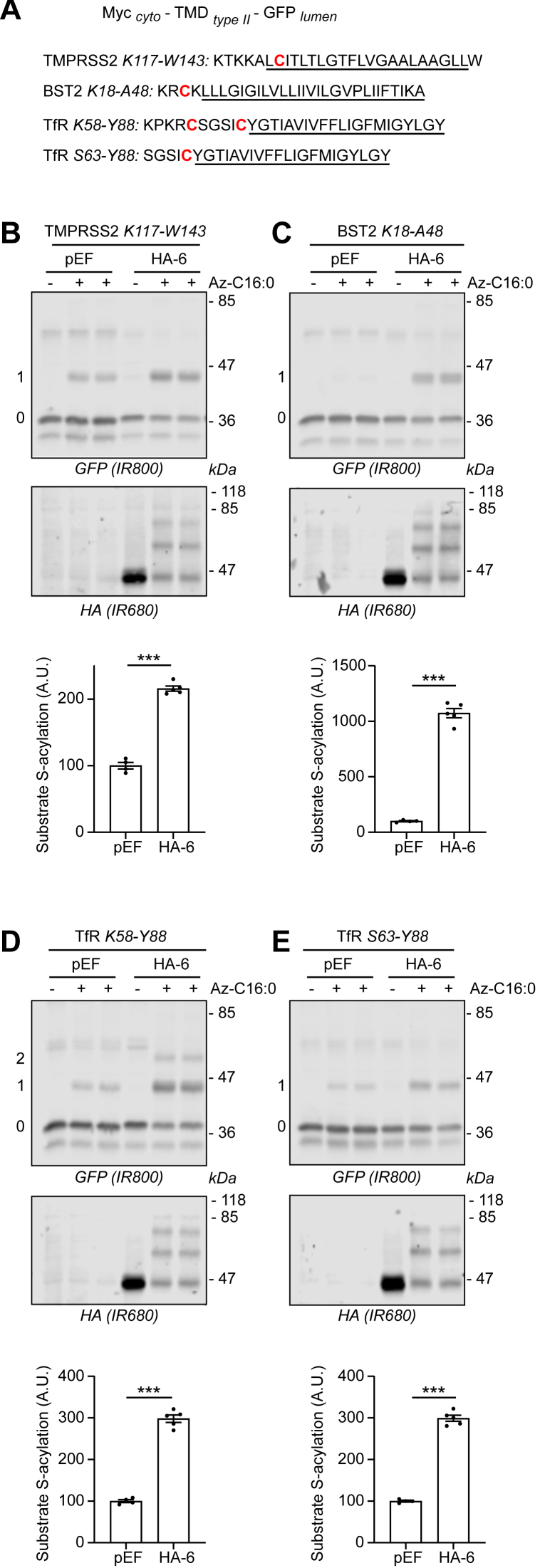
**A minimal transmembrane domain (TMD) of type II transmembrane proteins is sufficient for the S-acylation of their proximal cysteines by zDHHC6.***A*, schematic of the constructs used in the analysis. A small Myc cytoplasmic tag (EQKLISEEDL) was fused at the N-terminal part of the TMDs (and proximal region) of several type II transmembrane proteins; the theoretical region spanning the bilayer is *underlined*. A GFP tag was fused at the C-terminal part of the constructs following the transmembrane segments. Type II proteins chosen here are TMPRSS2, BST2, and transferrin receptor (TfR). *B*–*E*, HEK293T cells were cotransfected with a plasmid encoding each of the constructs described in *A* together with a plasmid encoding HA-DHHC6 (HA-6) or the empty control plasmid (pEF). Six hours post-transfection, cells were metabolically labeled overnight with either palmitate (−Az-C16:0) or palmitate azide (+Az-C16:0) followed by click chemistry using alkyne-PEG. Proteins were resolved by SDS-PAGE and transferred to nitrocellulose membranes. The membranes were probed first with an anti-GFP antibody (revealed with secondary antibodies coupled to the infrared dye IR800) followed by an anti-HA antibody (and IR680-coupled secondary antibodies). Positions of molecular weight markers (in kilodalton) are shown on the *right-hand side* of the membranes, whereas numbers on the *left* (0, 1, and 2) relate to the number of modified cysteines within the transmembrane fusion proteins. The *graph* below each *panel* shows mean ± SEM of normalized substrate S-acylation by HA-tagged DHHC6; *filled circles* represent individual samples (n = 4–5 cell samples from two independent experiments). Statistical analysis (Student’s *t* test) was performed to reveal significant S-acylation of the substrates following the expression of exogenous HA-DHHC6 *versus* its acylation by endogenous enzymes (pEF samples) (∗∗∗*p* < 0.001). BST2, bone marrow stromal antigen 2; HA, hemagglutinin; HEK293T, human embryonic kidney 293T cell line; TMPRSS2, transmembrane serine protease 2; TfR, transferrin receptor.

To examine the features in TMPRSS2 and BST2 that are recognized by zDHHC6, we generated truncation mutants. Strikingly, we found in all cases that the TMD and adjacent cytosolic basic amino acids (which for BST2 surround the target cysteine) were sufficient for S-acylation by zDHHC6 (Fig. 6, *A*–*C*). We also examined if this is the case for the transferrin receptor (TfR), which like BST2 and TMPRSS2 is a type II membrane protein that has been shown to be modified by zDHHC6 (25, 26). Two TfR constructs were examined, one that contained the TMD and a single adjacent cytosolic cysteine (S63–Y88) and another that contained two cysteines (K58–Y88) (Fig. 6*A*). Figure 6, *D* and *E* shows that both constructs were effectively S-acylated by zDHHC6; for the K55–Y88 construct two band shifts are visible showing that this truncation mutant is S-acylated at both cysteines.

**Figure 7:**
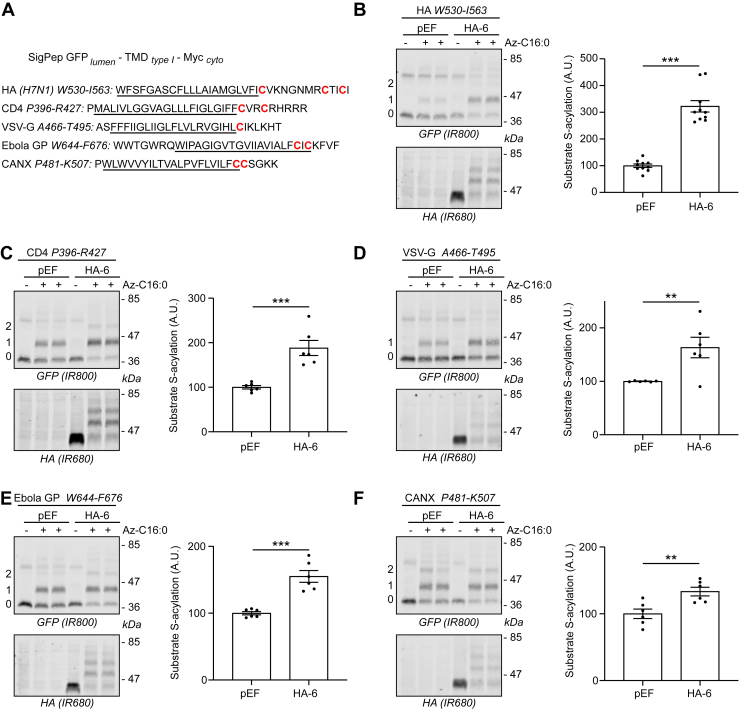
**Proximal cysteines of the transmembrane domains of type I membrane proteins can also be S-acylated by zDHHC6.***A*, schematic of the constructs used in the analysis. The signal peptide (SigPep) of azurocidin was inserted upstream of the coding sequence of GFP to allow for its translocation into the lumen of the endoplasmic reticulum. The transmembrane domains and proximal cysteines of several type I membrane proteins were fused to GFP at their N terminus and to the Myc tag (EQKLISEEDL) at their C terminus. The theoretical regions spanning the bilayer are *underlined*. Type I proteins chosen here are influenza (H7N1) hemagglutinin, cluster of differentiation 4 (CD4), the glycoprotein of the vesicular stomatitis virus (Indiana strain) (VSV-G), Ebola glycoprotein (Ebola GP), and human calnexin (CANX). *B*–*F*, HEK293T cells were cotransfected with a plasmid encoding each of the constructs described in *A* together with a plasmid encoding HA-DHHC6 (HA-6) or the empty control plasmid (pEF). Six hours post-transfection, cells were metabolically labeled overnight with either palmitate (−Az-C16:0) or palmitate azide (+Az-C16:0) followed by click chemistry using alkyne-PEG. Proteins were resolved by SDS-PAGE and transferred to nitrocellulose membranes. The membranes were probed first with an anti-GFP antibody (revealed with secondary antibodies coupled to the infrared dye IR800) followed by an anti-HA antibody (and IR680-coupled secondary antibodies). Positions of molecular weight markers (in kilodalton) are shown on the *right-hand side* of the membranes, whereas numbers on the *left* (0, 1, and 2) relate to the number of modified cysteines within the transmembrane fusion proteins. The *graph* on the *right-hand side* of each *panel* shows mean ± SEM of normalized substrate S-acylation by HA-tagged zDHHC6; *filled circles* represent individual samples (n = 6–10 different cell samples from two independent experiments). Statistical analysis (Student’s *t* test) was performed to reveal significant S-acylation of the substrates following the expression of exogenous HA-DHHC6 *versus* its acylation by endogenous enzymes (pEF samples) (∗∗∗*p* < 0.001; ∗∗*p* < 0.01). HA, hemagglutinin; HEK293T, human embryonic kidney 293T cell line.

In addition, we tested if zDHHC6 is also able to S-acylate constructs containing only the TMD and adjacent cytosolic residues from type I membrane proteins (Fig. 7*A*). For this, we examined five different type I proteins that are known to be S-acylated: calnexin (CANX) (27, 28), cluster of differentiation 4 (CD4) (29), the glycoproteins (GPs) of Ebola (30) and the vesicular stomatitis virus (VSV-G) (31), and the influenza A H7N1 hemagglutinin protein (HA) (32, 33, 34) (Fig. 7*A*). Only CANX has been shown experimentally to be S-acylated by zDHHC6 (27). The identities of the zDHHC enzymes modifying CD4, VSV, and Ebola fusion proteins are unknown, whereas HA have been shown to be S-acylated by several enzymes (zDHHC2, 8, 15, and 20) but not by zDHHC6 (35). The inclusion of these constructs allowed us to broaden our search for potential specific properties within natural TMDs that would be recognized by zDHHC6. Their common feature is the presence of one or several cysteines in the vicinity of the TMD. As shown in Figure 7, zDHHC6 cotransfection increased the S-acylation of all five type I constructs, confirming that this enzyme is active against both type I and II membrane proteins.

**Figure 8:**
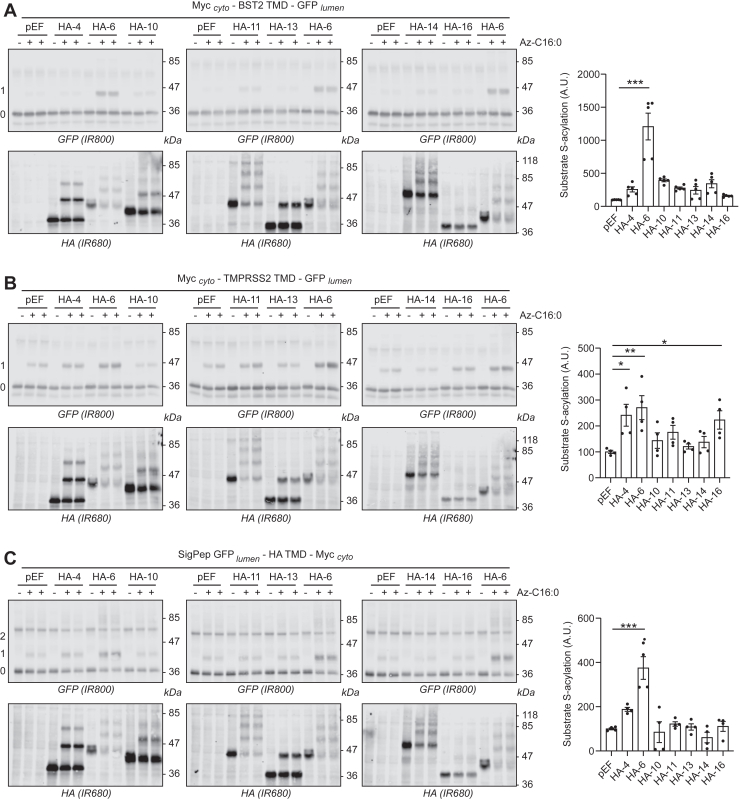
**S-acylation of proximal cysteines by selected endoplasmic reticulum–resident zDHHC enzymes.** HEK293T cells were cotransfected with a plasmid encoding GFP-tagged constructs of the transmembrane domains of type II (BST2 [*A*] and TMPRSS2 [*B*]) or a type I (HA [*C*]) membrane protein together with the empty control plasmid (pEF) or plasmids encoding selected HA-tagged DHHC enzymes (HA-4, -6, -10, -11, -13, -14, or -16). Six hours post-transfection, cells were metabolically labeled overnight with either palmitate (−Az-C16:0) or palmitate azide (+Az-C16:0) followed by click chemistry using alkyne-PEG. Proteins were resolved by SDS-PAGE and transferred to nitrocellulose membranes. The membranes were probed first with an anti-GFP antibody (revealed with secondary antibodies coupled to the infrared dye IR800) followed by an anti-HA antibody (and IR680-coupled secondary antibodies). Positions of molecular weight markers (in kilodalton) are shown on the *right-hand side* of the membranes, whereas numbers on the *left* (0, 1, and 2) relate to the number of modified cysteines within the transmembrane fusion proteins. The *graph* on the *right-hand side* of each *panel* shows mean ± SEM of normalized substrate S-acylation by HA-tagged zDHHC enzymes; *filled circles* represent individual samples (n = 4 different cell samples from two independent experiments). Statistical analysis (Student’s *t* test) was performed to reveal significant S-acylation of the substrates following the expression of exogenous HA-DHHC enzymes *versus* its acylation by endogenous enzymes (pEF samples) (∗∗∗*p* < 0.001; ∗∗*p* < 0.01; and ∗*p* < 0.05). BST2, bone marrow stromal antigen 2; HA, hemagglutinin; HEK293T, human embryonic kidney 293T cell line; TMPRSS2, transmembrane serine protease 2.

Finally, to ensure that isolated TMDs do not have loose enzyme selectivity, we tested the ability of six other ER-localized zDHHC enzymes to modify the TMD of BST2, TMPRSS2, and HA. As shown in Figure 8, *A* and *C*, only zDHHC6 expression induced a significant increase in the S-acylation of the BST2 and HA TMDs. In contrast, the TMD of TMPRSS2 did display a more diverse pattern of enzyme recognition/selectivity (Fig. 8*B*). Specifically, both zDHHC4 and zDHHC16 caused a similar increase of TMPRSS2 TMD S-acylation as zDHHC6, whereas these enzymes were not active against full-length TMPRSS2 (Fig. 1), suggesting that this transmembrane had a looser selectivity than the full-length protein.

## Discussion

We have previously proposed that the Golgi contains zDHHC enzymes with markedly different properties (14). Whereas, zDHHC17 appears to recognize specific features in substrate proteins to facilitate their S-acylation, analysis of zDHHC3 and zDHHC7 failed to identify a specific mechanism of substrate recognition (14, 16, 17). Indeed, it is fair to say that these enzymes are able to S-acylate the large majority of proteins analyzed in coexpression assays. Based on this, we have suggested that these enzymes be classified as high-activity/low-specificity enzymes and that their broad substrate selectivity profile stems from an intrinsic high activity that allows them to transfer the acyl chain (acquired through enzyme autoacylation) to any accessible cysteine in membrane proximity. In this model, S-acylation specificity is determined by the presence of a suitably positioned cysteine that is accessible for S-acylation. In this way, a large and diverse pool of cellular proteins trafficking though the secretory pathway can be S-acylated without the requirement for specific modes of enzyme–substrate interaction. Indeed, as recent work suggested that S-acylation is required for Golgi exit of transmembrane proteins, this loose enzyme specificity fits with a model in which only S-acylated proteins leave the Golgi, negating the need for high-affinity interactions (9). We propose that the higher affinity substrate interactions mediated by the likes of zDHHC17 are more important in recruiting soluble proteins to the Golgi to facilitate their S-acylation at this compartment and allow directional movement to the plasma membrane and endocytic compartments.

The work presented in this article suggests that broad specificity enzymes are also localized at the ER. We identify zDHHC6 as a major ER-resident and broad specificity enzyme that is capable of S-acylating reactive cysteines proximal to or within a TMD. zDHHC6 was indeed capable of modifying all eight transmembrane constructs tested, regardless of their orientation and amino-acid composition. The only feature of full-length TMPRSS2 and BST2 that appeared to be important for their S-acylation by this enzyme was the close position of the cysteine relative to the TM domain. The target cysteine of TMPRSS2 is situated within its theoretical transmembrane region. Our finding that moving the modified cysteine in TMPRSS2 to a position five amino acids into the TMD (from the cytosolic region) ablated S-acylation by zDHHC6 is consistent with findings from Rodenburg *et al.* (36) who showed by native mass spectrometry that cysteines situated up to 8 Å into the inner leaflet of the membrane in integral proteins were modified by S-acylation. We propose that zDHHC6 might therefore mediate S-acylation of multiple membrane proteins in the ER relying on an intrinsic high S-acylation activity (as suggested by Abrami *et al.* (25)) and weak (low specificity) interactions between transmembrane regions of the enzyme and substrate proteins.

Some of the transmembrane constructs screened here were based on proteins previously known to be S-acylated by zDHHC6 (*i.e.*, CANX (27) and TfR (26)). We also identify two novel zDHHC6 substrates, BST2 and TMPRSS2. It is tempting to speculate that CD4 and the GPs of VSV and Ebola are also substrates of zDHHC6, based on the results obtained with their respective transmembrane constructs. The identity of the zDHHC enzymes that S-acylate these proteins is still unknown (29, 33, 34, 37). On the other hand, zDHHC6 was not detected in an siRNA screen aimed at identifying HA S-acylating enzymes; instead, the depletion of zDHHC2, 8, 15, and 20 was the most effective at reducing HA S-acylation (35). HA displays a very short cytoplasmic region containing three cysteines (C551, 559, and 562), one of them only (C551) being at an ideal position of +1 in regard to the transmembrane, whereas the two others are further away (positions +9 and +12; Fig. 7*A*). Our mPEG click chemistry assay detects one main band shift, suggesting that the first cysteine might indeed be the main residue modified by zDHHC6 (Fig. 7*B*). Interestingly, C551 was identified as exclusively stearoylated (covalent addition of the saturated C18:0 lipid) rather than palmitoylated (32). Stearoylation is a property shared with TfR, another substrate of zDHHC6, as well as with zDHHC6 itself (26). It will be interesting to investigate the acyl chain specificity of zDHHC6 and if the enzyme has an intrinsic preference for longer chain fatty acids.

Among the few experimentally established substrates of zDHHC6, many are either type I or type II transmembrane proteins: CANX (25, 27), TfR (25, 26), the interferon-induced transmembrane protein 3 (IFITM3) (38), the tight junction protein angulin-1 (39), or the recently identified substrates AEG1 and CLIMP-63/CKAP4 (40, 41). All these proteins display one or several cysteines in very close proximity to their TMD. The vicinity of the S-acylated cysteines to the two transmembrane segments of CD36 is compatible with their S-acylation by zDHHC6 (42, 43). In contrast, a few substrates of zDHHC6 can be either cytosolic proteins (such as NRas (44) or Myd88 (45)) or display cysteines that are very distal from any TMD (such as inositol 1,4,5-triphosphate receptor (46) or AMFR/gp78 (25, 47)). This suggests that, in addition to the broad and potentially loose recognition of TMDs by zDHHC6 that is uncovered in this study, there might be a more specific recognition of some substrates through their cytosolic domains. Nevertheless, the S-acylation of cytosolic proteins does not appear to be a general feature of zDHHC6 (as opposed to zDHHC3 and zDHHC7); indeed many cytosolic proteins (*e.g.*, such as SNAP25, CSP, or PSD95) cannot be S-acylated following coexpression of zDHHC6 (4, 12).

Acylation of cytosolic cysteines in close proximity to transmembrane segments has long been recognized as a common occurrence among S-acylated proteins (34, 36, 48, 49). Strikingly, none of the six other ER-resident zDHHC enzymes tested here were able to S-acylate the constructs containing the TMDs of BST2 or HA, apart from zDHHC6, which suggests that the latter might be one of the main enzymes performing this task in the ER. Full-length BST2 was more efficiently S-acylated (than its corresponding transmembrane construct) by zDHHC4 and zDHHC11 (HA-4 and HA-10), suggesting that the cytosolic and extracellular domains might be part of a recognition process by these two enzymes. zDHHC11 (HA-10) was also capable of S-acylating full-length TMPRSS2 but not the corresponding transmembrane construct. In contrast to BST2, a TMPRSS2 TMD construct was as efficiently S-acylated by zDHHC4 and zDHHC16 as by zDHHC6, showing that other ER-resident enzymes can also recognize isolated transmembrane regions but maybe with a degree of specificity (since they do not affect BST2 or HA TMD S-acylation). The physicochemical properties of the different membrane-spanning segments might explain this observation.

A striking feature of zDHHC6 is its SH3 domain (a domain recognizing proline-rich sequences and mediating protein–protein interactions) featured at the C-terminal cytoplasmic tail of the enzyme. Our analysis suggests that the SH3 domain may not be important for the recognition of the eight TMD constructs examined as part of this study as they do not contain proline-rich sequences on the cytoplasmic side of the membrane. Thus, we propose that the SH3 domain is not essential for substrate S-acylation by zDHHC6 although it could be important for modification of some substrates. Alternatively (or in addition), zDHHC6 SH3 domain has been found to bind selenoprotein K (SelK) (46, 50), a possible cofactor of zDHHC6. SelK appears to act by preventing the premature hydrolysis of the acyl-zDHHC6 intermediate that is generated within the active site of the enzyme during the ping–pong S-acylation reaction. A zDHHC6–SelK complex has been implicated in the S-acylation of the inositol 1,4,5-triphosphate receptor (46) and the scavenger receptor/lipid transporter CD36 (42, 43), but evidence for the involvement of SelK in the S-acylation of other substrates is currently missing.

Of note, there is a very low (almost undetectable) level of S-acylation of FLAG-tagged full-length BST2 in the absence of coexpressed enzyme, which is in accordance with data previously published by our group, which at the time led us to conclude that BST2 was not an acylated protein (51). It is interesting that S-acylation of the proteins (especially BST2) was very low in the absence of zDHHC enzyme coexpression. It has been difficult to study the protein expression of endogenous zDHHC enzymes in detail, which is thought to be due to their low expression levels and a lack of sensitive antibodies. Thus, overexpression of substrate proteins may saturate the endogenous S-acylation machinery and require coexpression of enzymes to allow detectable S-acylation. Although we do not have a clear picture of the relative expression levels of zDHHC enzymes in HEK293T cells, previous work using quantitative PCR analysis suggested that mRNA encoding most of the zDHHC isoforms (including zDHHC6) was detectable in HEK293 cells. The level of zDHHC4 mRNA was the highest in this cell line and approximately 5× higher than zDHHC6 mRNA levels (52).

S-acylation has been described to affect the stability, trafficking, and interactions of many different proteins (1). At present, we do not know how S-acylation affects these parameters for BST2 and TMPRSS2. Although this will be the focus of future work, some speculation can be provided here. TMPRSS2 is a protease of the type II transmembrane serine protease family that is synthesized as a single-chain zymogen N-glycosylated form that becomes active following self-cleavage in the Golgi apparatus (53). TMPRSS2 autoactivation is negatively regulated by binding to specific cellular inhibitors such as plasminogen activator inhibitor-1 (SERPIN E1) (54) or hepatocyte growth factor activator inhibitors HAI-1 and HAI-2 (53, 55). TMPRSS2 S-acylation could potentially impact these protein interactions, its trafficking through the secretory pathway, and/or its protease activity. Substrates of TMPRSS2 include angiotensin-converting enzyme 2 (56, 57, 58) (one of the cellular receptors for SARS-CoV-2 (19)) and viral proteins such as the influenza HA (54) and coronavirus spikes (19, 56, 59). TMPRSS2 inactivation in mice was shown to increase their resistance to several strains of influenza and to SARS-CoV-2 infection (60, 61, 62, 63). TMPRSS2 is also central to prostate cancer metastasis and constitutes a target for treatment (55, 64). The identification of S-acylation as a novel post-translational modification of TMPRSS2 could therefore also lead to the exploration of this pathway for therapeutic intervention.

BST2, on the other hand, is a broad, interferon-induced, antiviral protein renamed “tetherin” to illustrate its ability to inhibit retrovirus release from infected cells (22, 65, 66). BST2 displays a very unusual topology (shared with the prion protein PrPc): it possesses both an N-terminal TMD and a C-terminal glycosylphosphatidylinisotol anchor (67). BST2 associates with the cortical actin cytoskeleton (68) and organizes membrane lipid microdomains (67, 69). BST2 has emerged as a key player in the host cell defence against a variety of enveloped viruses, including the lentiviruses HIV-1 and HIV-2, the herpesvirus HHV-8, the filoviruses Ebola and Marburg and many others (reviewed in Ref. (70)). As a consequence of its broad antiviral activity, BST2 is a target for inactivation by most enveloped viruses. Indeed, viruses have evolved a variety of mechanisms to counteract BST2 restrictive activities, leading in general to its degradation or sequestration through various cellular pathways (reviewed in Ref. (21)). Beyond its direct role in the restriction of viral dissemination, BST2 acts as a viral sensor by inducing NF-κB-dependent proinflammatory responses to HIV-1 infection (23, 71, 72). In addition, BST2 prevents the detrimental sustained activation of RIG-I-like receptor–mediated type I interferon signaling by providing a negative feedback loop (73). Any of the aforementioned properties of BST2 could potentially be affected by its S-acylation as a result of, for example, changes in cellular localization or interactions with cellular and viral partners.

In conclusion, our data reveal that the ER resident zDHHC6 is capable of S-acylating any cysteine in the vicinity of the TMD of several type I and type II proteins. We propose that, similar to Golgi-localized zDHHC3 and zDHHC7, ER-localised zDHHC6 is a broad specificity/high-activity S-acylating enzyme.

## Experimental procedures

### Antibodies

Mouse GFP antibody (Clontech; clone JL8, used at 1:4000 dilution) was obtained from Takara. Rat HA tag antibody (Roche; clone 3F10, used at 1:1000 dilution) was from Merck. Anti-FLAG antibodies were from Merck (rabbit clone F7425, used at 1:1000 dilution) and GenScript (THE DYKDDDDK mouse antibody, used at 1:1000 dilution). IR dye–conjugated secondary antibodies (used at 1:20,000 dilution) were purchased from LI-COR Biosciences.

### Plasmid DNA

The complementary DNA (cDNA) encoding all the mouse enzymes was subcloned into pEF-BOS HA by Fukata *et al.* (4). The numbering of the enzyme isoforms is according to Fukata *et al.* (4). The cDNAs encoding the long isoform of human TMPRSS2 (NM_001135099.1; catalog number: OHu13718D) (in fusion with a FLAG tag at its C terminus) and BST2 (NM_004335.4; catalog number: OHu15823) were obtained from GenScript. BST2 was subsequently amplified by PCR and subcloned between the XhoI and XbaI sites of the pCS2 FLAG plasmid (Addgene #16331) in order to fuse a FLAG tag at the N terminus of BST2. Site-directed mutagenesis was used to introduce cysteine mutations within these plasmids by using oligonucleotide primers designed with the “quick change primer design” online tool from Agilent and synthesized by Merck. PCR was performed using Pfu polymerase (catalog no.: M7741; Promega). The theoretical boundaries of the amino acids spanning the bilayer were obtained either from the UniProt or National Center for Biotechnology Information websites. The plasmids encoding the type II transmembrane constructs Myc-TMPRSS2 K117_W143-GFP, Myc-BST2 K18_A48-GFP, Myc-TfR K58_Y88-GFP (TfR: UniProt ID: P02786), and Myc-TfR S63_Y88-GFP were synthesized and cloned into pcDNA3.1(+)-C-eGFP by GenScript. The plasmids encoding the type I transmembrane constructs were built by annealed oligonucleotide cloning with long oligonucleotides synthesized by Merck. First, the cDNA encoding the signal peptide of the azurocidin 1 preprotein (NM_001700.5, amino acids 1–19) (74) was inserted in 5′ of the coding sequence of eGFP within pEGFP-C2 plasmid (Clontech). The coding sequence of the Myc tag (EQKLISEEDL) was then inserted at the 3′ end of the multiple cloning site of the vector. Finally, the TMDs of human CD4 (NM_000616.5/UniProt ID: P01730.1), human CANX (NM_001024649.2/UniProt ID: P27824.2), influenza A (H7N1) HA (GenBank number: ACZ48602.1), Zaire Ebolavirus GP (NC_002549.1), and VSV GP (NC_001560.1/UniProt ID: P03522) were inserted in fusion with GFP and Myc within the multiple cloning site of the modified eGFP-C2 plasmid.

The validity of all constructs was confirmed by sequencing (Dundee DNA Sequencing Service).

### Cells

HEK293T cells (CRL-3216; American Type Culture Collection) were grown in Dulbecco’s modified Eagle’s medium (Thermo Fisher Scientific) supplemented with 10% fetal bovine serum at 37 °C in a humidified atmosphere containing 5% CO_2_.

### Cell transfection and labeling with fatty acids

HEK293T cells were plated on poly-d-lysine–coated 24-well plates (Corning BioCoat; VWR) and transfected with 0.33 μg of substrate plasmid and 0.66 μg of pEF-BOS-HA (pEF) plasmid (either empty as a control or encoding the DHHC enzymes). Two μl of polyethylenimine (1 mg/ml stock) (linear polyethyleneimine molecular weight 25,000, catalog no.: 43896; Alpha Aesar) was added to the DNA mix, incubated for 20 min, and then added to the cells. Six h post-transfection, cells were washed once with PBS and incubated overnight with 100 μM of either palmitic acid (P0500; Merck) or C16:0-azide (75) in 300 μl of serum-free Dulbecco’s modified Eagle’s medium supplemented with 1 mg/ml defatted bovine serum albumin (A7030; Merck) at 37 °C.

### Detection and quantification of S-acylation by PEG click chemistry

The cells were washed once with PBS and then lysed in 100 μl of 50 mM Tris, pH 8.0, containing 0.5% SDS and protease inhibitors (P8340; Merck). Conjugation of mPEG5K-Alkyne to C16:0-azide was carried out for 1 h at room temperature with end-over-end rotation by adding an equal volume (100 μl) of freshly prepared click-chemistry reaction mixture containing the following: 200 μM mPEG5k-Alkyne (JKA3177; Merck), 4 mM CuSO_4_ (catalog no.: 451657; Merck), 400 μM Tris[(1-benzyl-1*H*-1,2,3-triazol-4-yl)methyl]amine (catalog no.: 678937; Merck), and 8 mM ascorbic acid (catalog no.: A15613; Alpha Aesar) in distilled water. About 70 μl of 4× SDS sample buffer containing 100 mM DTT was then added to the 200 μl sample. Protein samples were incubated at 95 °C for 5 min, and 20 μl was resolved by SDS-PAGE and transferred to nitrocellulose for immunoblotting analysis. Immunoblots were scanned with an Odyssey LI-COR infrared scanner and quantified with the ImageStudio software (LI-COR). The efficiency of S-acylation was calculated as the ratio between the sum of signals corresponding to the modified cysteines and the sum of all the signals (*i.e.*, including the unacylated form). The efficiency of substrate acylation by different enzymes was normalized to control conditions without any coexpressed enzyme (*i.e.*, with pEF). To compare the acylation of mutant substrates *versus* wildtype substrates, the efficiency of acylation in control conditions without any coexpressed enzyme (*i.e.*,with pEF) was subtracted first. Then the acylation of mutant substrates in cells coexpressing HA-DHHC6 was normalized to the value of acylation of the wildtype substrate in the same conditions. Graphs were created, and statistical analysis was performed using GraphPad software (GraphPad Software, Inc).

## Data availability

All data are contained within the article.

## Conflict of interest

The authors declare that they have no conflicts of interest with the contents of this article.
